# Elemental Profile in Chicken Egg Components and Associated Human Health Risk Assessment

**DOI:** 10.3390/toxics11110900

**Published:** 2023-11-03

**Authors:** Cezara Voica, Gabriela Cristea, Andreea Maria Iordache, Carmen Roba, Victor Curean

**Affiliations:** 1 National Institute for Research and Development of Isotopic and Molecular Technologies, 400293 Cluj-Napoca, Romania; cezara.voica@itim-cj.ro; 2 National Research and Development Institute for Cryogenics and Isotopic Technologies, ICSI, 240050 Ramnicu Valcea, Romania; andreea.iordache@icsi.ro; 3 Research Department, Faculty of Environmental Science and Engineering, Babes-Bolyai University, 400294 Cluj-Napoca, Romania; carmen.roba@ubbcluj.ro; 4 Faculty of Pharmacy, “Iuliu Hatieganu” University of Medicine and Pharmacy, 400012 Cluj-Napoca, Romania

**Keywords:** egg, metals, health risk, chemometrics

## Abstract

Egg is a food product of high nutritional quality, extensively consumed worldwide. The objectives of this study were the determination of the elemental profile in eggs (egg white, yolk, and eggshell), the estimation of the non-carcinogenic health risk associated with the presence of heavy metals in investigated egg samples, and the development of statistical models to identify the best predictors for the differentiation of egg components. The assessments were carried out in a total set of 210 samples, comprising home-produced and commercial eggs, using inductively coupled plasma mass spectrometry. The results suggested measurable differences amongst hen eggs coming from different husbandry systems. The statistical models employed in this study identified several elemental markers that can be used for discriminating between market and local producer samples. The non-carcinogenic risk related to the consumption of the analyzed egg samples was generally in the safe range for the consumers, below the maximum permitted levels set by Romanian and European legislation. Food contamination is a public health problem worldwide, and the risk associated with exposure to trace metals from food products has aroused widespread concern in human health, so assessing the heavy metal content in food products is mandatory to evaluate the health risk.

## 1. Introduction

Maintaining food safety has become a significant challenge in food production, consumption, and management, and worldwide attention to food safety has increased. Thus, food quality and safety must be a constant concern both for consumers and producers [1].

The poultry industry is one of the largest sectors of agriculture throughout the world, and the selection for egg quality is an essential component of the breeding strategies of companies that market egg-laying type hens [2,3,4]. Consumers demand high-quality products with solid eggshells while reducing cost, guaranteeing eggs devoid of contaminants and improving the acceptability of rearing systems. Therefore, most selection strategies to enhance the quality of the eggs have focused on the shell’s physical properties and stability of egg weight [5,6].

Chicken eggs are one of the main sources of protein but, if contaminated by toxic heavy metals, become a problem for environmental and human health [7]. Poultry can take up heavy metals from different sources, primarily via nutrition, so metal residues may concentrate in their eggs [8,9,10]. Chicken eggs are considered one of nature’s highly nutritious food items in the human daily diet, being highly responsible for human health [11]. Because most chickens are reared on farms, where a range of feed additives are used, concerns have been raised about the potential public health risk of chicken product consumption [12]. The human health risk assessment requires identifying, collecting, and integrating information on hazardous chemicals, their exposure to humans, and the relationship between exposure, dose, and adverse health effects [13].

Members of different income classes generally consume eggs. They are low-cost, nutrient-dense foods, providing 6.3 g of protein distributed between the yolk and white portions (3.6 g in egg white and 2.7 g in egg yolk) and they are low in saturated fat relative to other sources of high-quality protein [14]. Eggs contain several nutritional components, which protect against chronic disease, including lutein, zeaxanthin, choline, vitamin D, selenium, and vitamin A. In addition, eggs are considered to be one of the best dietary sources of high-quality protein, with a very high nutritional value due to their rich content of essential amino acids, which is why the World Health Organization has chosen the egg as a reference standard for assessing the quality of proteins from other animal products. Eggs, meat, and dairy are digested at a rate above 90%, compared to a range of 45–80% for plant proteins [15]. Of the three basic foods, eggs have been reported to be the most digestible protein source by the World Health Organization, measured as 97% [16]. Net protein utilization (NPU) is an index of protein quality calculated by multiplying protein digestibility by biological value. The NPU of grains is generally less than 40 (rice is the exception, with an NPU of about 60, but it is low in protein (7.5 percent); the NPU of chicken eggs is 87) [17]. Eggs contain all nine essential amino acids, making them a complete protein, so the ratio and pattern in which these amino acids are found make them the perfect match for the body’s needs [18,19,20]. Besides proteins, eggs are important sources of high-quality fats (lecithin) and are rich in unsaturated fatty acids, so eggs protect against infection, act as a hypotensive agent, and even protect against cancer [21].

Eggs belong to the products covered by EU regulation 1308/2013 on the typical organization of the agricultural markets [22]. The EU supports egg producers through marketing standards and occasionally through some market support measures [23]. The egg market has radically changed due to branding and the introduction of quality marking and the visible inscription of the quality mark, with a well-defined brand identity. So, the producers who desire differentiation and quality have led the market to a much higher level, both in terms of the added value of the actual product and its image. More than 350 million laying hens are registered in the European Union, with an annual production of approximately 6.7 million tonnes of eggs [23]. Romania is among the first countries in the European Union regarding the small number of hens raised in cages. Out of over 32 million laying hens, only a quarter belong to specialized farms, and the rest belong to individual households. The annual domestic production is 5.4–5.5 billion eggs from rural households (around 60%) [24]. Romanian consumers have become more and more increasingly aware of health issues and in purchasing sustainable products of better quality. One of the most essential criteria for egg selection is the conditions in which the chickens were raised. In terms of perception and attitude, current consumers have an orientation toward fresh, possibly organic products, also stimulated by the new legislative regulations that require more information to be marked on food products [25]. “Home-grown” production of a certain food product (meat, fruits, eggs, milk) is seen as a clean and green alternative as compared to commercial production systems [26]. Occasionally, residential yards are not necessarily clean environments, and yard soils may contain contaminated residues, creating the potential for exposure to various contaminants [27]. Some studies demonstrated the potential for the bioaccumulation of a range of pollutants in chickens’ tissues and eggs [28,29].

There are diverse studies using different analytical methods for egg investigation: gas chromatography-mass spectrometry [30]; high-performance liquid chromatography [31]; hyperspectral imaging [32]; elemental analysis [26,33]; near-infrared spectroscopy [34]; and isotope ratio mass spectrometry—IRMS [35,36,37]. Among these analytical techniques, mass spectrometry takes the leading place, being recognized at the EU level as an important tool in the authentication and traceability of food products [38,39,40,41]. There are few studies carried out on hen eggs based on determining the heavy metal contamination of poultry eggs and the adverse effect of their consumption on human health and risk assessment [42,43,44,45,46,47,48].

Given the range of chicken and eggs available at the Romanian market, there is very little information on the heavy metal concentration in hen eggs, or the comparison of the contamination level of toxic metals between poultry eggs and household/domestic eggs. The Romanian food system heavily depends on egg or meat chickens, and their contamination can have disastrous effects; therefore, this study was conducted with a special look at hen eggs as one of the most Romanian consumed foods. Egg quality was assessed to establish the possible risk to human health using heavy metals concentrations, due to their cumulatively negative impact on human health.

Consequently, this study (i) determined the elemental profile (macro- and microelements, metals with toxic potential, rare earth elements) of egg components (egg white, yolk, and eggshell) using Inductively Coupled Plasma—Mass Spectrometry (ICP-MS); (ii) estimated the non-carcinogenic health risk associated with the presence of heavy metals in investigated egg samples; and (iii) developed statistical models, based on eggs’ elemental concentrations, to identify the best predictors for the differentiation of egg components and also the differentiation of the rearing system of hens from where the eggs come from (commercial versus yard).

## 2. Materials and Methods

### 2.1. Egg Collection and Sample Preparation

A total set of 70 hen eggs, totaling 210 samples (egg white, *n* = 70; egg yolk, *n* = 70; and eggshell, *n* = 70), was analyzed using Inductively Coupled Plasma—Mass Spectrometry in order to obtain their elemental fingerprints. From the entire set, 55 eggs were collected from local producers, coming from backyard growing systems, and the remaining 15 eggs came from the supermarket, originating from commercial rearing systems.

In the sample preparation process, the eggshells were washed with acetone and deionized water to remove adherent external pollution [49]. Then, the egg components (egg white, yolk) and eggshells were placed in an oven to achieve a constant dry weight. In the next step, all dried samples were homogenized individually. The total digestion of the matrix is mandatory to assure complete metal solubility, knowing that the samples have a very complex composition with significant organic matter content. Thus, 0.5 g of each sample with 10 mL of 65% ultrapure HNO_3_ was placed in a clean Teflon digestion vessel. For microwave-assisted digestion, a microwave reaction system (MWS-2, Microwave oven speed wave, Berghof, Germany) was used, programmable for time and power, having 20 high-pressure polytetrafluoroethylene vessels. After this, the system was cooled at room temperature, and the contents of the tubes were transferred to 50 mL self-standing polypropylene volumetric tubes diluted with ultrapure deionized water (18 MΩ·cm^−1^), from a Milli-Q analytical reagent grade water purification system (Millipore, Darmstadt, Germany), in order to continue multi-element analyses.

An inductively coupled plasma mass spectrometer (Perkin Elmer ELAN DRC-e, Norwalk, CT, USA) with a Meinhard nebulizer and a glass cyclonic spray chamber for pneumatic nebulization was used for all elemental measurements. The operating conditions were as follows: gas flow in nebulizer—0.92 L/min, auxiliary gas flow—1.2 JL/min, plasma gas flow—15 L/min, lens voltage—7.25 V, radiofrequency power—1100 W, CeO/Ce ratio—0.030, and Ba++/Ba ratio—0.029. In addition, Equation (1) was used for converting the concentration of heavy metals determined for acid-digested tissue solution (μg/L) to metal concentrations (μg/g) in samples (eggshells and egg components):

Ct = (Cs × Vs)/Wt,(1)

where Ct is the metal concentration in the tissue sample (μg/g); Cs is the metal concentration in the acid-digested solution (μg/L); Vs is the volume of acid-digested sample solution (L); and Wt is the dry weight of the sample (g) [50]. Metal concentration in the samples (eggshells and egg components) is expressed as μg/g on dry weight (dw).

### 2.2. Health Risk Assessment

The non-carcinogenic health risk associated with the presence of heavy metals in eggs can be estimated based on the method proposed by US-EPA [51], by quantifying the Target Hazard Quotient (THQ) according to Formula (2):

(2) $$\mathrm{THQ}=\frac{\mathrm{EF}\;*\;\mathrm{ED}\;*\;\mathrm{IR}\;*\;C}{\mathrm{BW}\;*\;\mathrm{AT}\;*\;\mathrm{RfD}}$$

where EF is the exposure frequency (365 days/year); ED is the exposure duration (75.88 years is the average lifetime for adults according to National Institute of Statistics [38]); IR is the average intake rate of food (0.0328 kg/person/day for eggs) [52]); C is the concentration of metal in eggs (mg/kg); BW is the average body weight of an individual (70 kg); AT is the average exposure time (365 days year × 75.88 years); RfD is the oral reference dose (RfD_Al = 1000 µg/kg/day, RfD_Cr = 1500 µg/kg/day, RfD_Mn = 140 µg/kg/day, RfD_Co = 43 µg/kg/day, RfD_Ni = 20 µg/kg/day, RfD_Cu = 40 µg/kg/day, RfD_Zn = 300 µg/kg/day, RfD_As = 0.304 µg/kg/day, RfD_Cd = 1 µg/kg/day, RfD_Sn = 0.3 µg/kg/day, RfD_Hg = 0.571 µg/kg/day, RfD_Pb = 3.57 µg/kg/day) [53].

By summing the THQ for each metal, the Total Target Hazard Quotient (TTHQ) was calculated to evaluate the cumulative potential health risk caused by exposure to the mixture of heavy metals. If the TTHQ ≤ 1, there is no risk, even for sensitive populations. At the same time, a value >1 indicates the probability of adverse health effects and suggests the need for further investigation or possible remedial actions.

### 2.3. Statistical Analysis

The statistical analysis was conducted using R 4.2.1 with the FactoMiner (version 2.9) and mixOmics (version 3.18) packages for multivariate data analysis [54,55,56]. This study comprised 70 egg samples, with 15 sourced from the supermarket and 55 sourced from local farms. Chemometric fingerprints were measured for each sample’s yolk, albumen, and eggshell, resulting in an initial dataset of 210 observations and 66 variables. Two discrete variables in the dataset indicate each sample’s egg part and rearing system of laying hen. In comparison, the remaining 64 variables measure the elemental composition in mg/kg of macro-minerals, heavy metals, toxic heavy metals, rare earth critical technological critical elements (TCEs), platinum group elements (PGEs), alkaline metals, alkaline earth metals, and transition- and post-transition metals. The dataset contains no missing values.

As a first exploratory step, principal component analysis (PCA) was conducted on the dataset, grouping the observations by egg component and growing system. The initial plot of the comments served as a mechanism for outlier detection. Upon identifying three outlier samples, they were removed and a new PCA was performed on the trimmed dataset.

Next, a Partial Least Squares Discriminant Analysis (PLS-DA) was performed with feature selection to differentiate samples based on (a) each egg part and (b) the laying hen’s growing system. PLS-DA is a statistical method commonly used for analyzing high-dimensional data with a categorical response variable and has been successfully applied in numerous chemometric analyses for discriminating various food products [57,58,59,60].

PLS-DA models the relationship between the predictors and the response variable by decomposing the predictor’s matrix into orthogonal components that explain the maximum covariance with the response variable. For this study, the following approach was used in both cases. Namely, an initial model was fitted using all variables with ten components to evaluate the performance and tune the final model. First, the ideal number of features was selected based on a 5-fold, 10-repeat cross-validation. Then, classification performance was assessed using the overall error rate (OER) and balanced error rate (BER) for three distance metrics (maximum, centroids, and Mahalanobis distance). After identifying the optimal number of components and variables, the data were shuffled. Then, we split them internally: 70% and 30% training and test sets, respectively, for evaluating the performance of the final, tuned model.

## 3. Results and Discussion

### 3.1. Elemental Determinations

The poultry industry has the fastest-growing agri-based production globally, with a more considerable significance for meat and eggs because they are good energy and protein sources [61]. In addition, the chicken egg naturally contains some metals and minerals. Mineral requirements of laying hens have to be met for optimum egg production, and minerals are needed for the formation of the skeletal system, for general health, as components of available metabolic activity, and for maintenance of the body’s acid–base balance [62].

Five essential elements (sodium (Na), magnesium (Mg), phosphorus (P), potassium (K), and calcium (Ca)) were explored in 70 eggs, totaling 210 samples, and the results for the mineral profile of eggs components (egg white, yolk, and eggshell) are presented in Table 1. There is a trend of mean concentrations in the order Na > K > P > Mg > Ca for egg white samples and in the order P > Ca > Na > K > Mg for yolk samples. The results obtained for egg components of the investigated samples are higher than those presented in other studies [63,64].

Calcium and phosphorus are the most abundant mineral elements in the human body. They are classified as macro-minerals, along with sodium, potassium, chloride, sulfur, and magnesium, being required in the diet at concentrations of more than 100 mg/kg. Phosphorus (P) is the third most expensive component of poultry feed and an essential macro-mineral nutrient for these, needed for the body growth, development of bones, and egg production. The imbalance in organic phosphorus sources in the diet mainly reduces poultry performance and health and increases the environmental pollution burden [65,66]. Generally, 60–80% of the total phosphorus present in plant-derived ingredients is in the form of phytate-phosphorus. The poultry birds cannot utilize the phytate-phosphorus of plant feedstuffs, because a considerable amount of P is present in the form of phytate, which is partially digested by chickens [67]. The low digestibility of phytate P means that inorganic phosphate, calcium phosphate, sodium phosphate, magnesium phosphate, and potassium phosphate are often added to the feed to meet dietary increments [68]. It is generally assumed that about one third of the phosphorus in plants and feedstuff is non-phytate and is biologically available to poultry, so the phosphorus requirement for poultry is expressed as non-phytate phosphorus rather than total phosphorus. A ratio of 2:1 must be maintained between calcium and non-phytate phosphorus in growing birds’ diets to optimize the absorption of these two minerals. The balance in laying birds’ diets is 13:1 because of the high requirement for calcium for good shell quality. Calcium and phosphorus are necessary for forming and maintaining the skeletal structure and good egg-shell quality. All living animals possess powerful mechanisms both to conserve calcium and to maintain constant cellular and extracellular concentrations, and these functions being so vital to survival that during severe dietary deficiency or abnormal losses of calcium from the body, they can demineralize bone to prevent even minor degrees of hypocalcemia [62].

The nutrition of laying hens, the characteristics of the feed, and the mode of delivery throughout the day affect the egg weight, yolk, and egg-white proportion [69]. The dietary characteristics include the level of calcium supply and its size. Dietary calcium in a particular form allows hens to express a specific appetite for calcium at the end of the day, which is stored and further assimilated during the night when the shell formation occurs [70]. The primary function of the eggshell is to protect the embryo from external aggression during its development. The shell constitutes about 10% of the egg content, representing 5 to 6 g per egg [71].

With an increase in the production of eggs by more than 150% in the past three decades, the resulting eggshell waste, which typically goes to landfills, poses severe hazards of environmental pollution and health [72,73]. However, it can be used as a valuable product, an attractive source of calcium for human nutrition, which could alleviate its environmental burden [74]. Eggshell is an inexpensive calcium source that is accessible at home, being composed of 94% calcium carbonate, 1% magnesium carbonate, and 1% of calcium phosphate [75]. Eggshell powder has excellent potential use as a calcium supplement in human nutrition, positively affecting bone human bone mass development.

Due to the great importance of some minerals and the properties of a hen’s egg, which is an essential food product, this present study was conducted to emphasize the content of some elements in hen eggs coming from two rearing systems, and to compare the results with other studies to underline the importance of consumer awareness on the food products consumed. The high average sodium (5431.04 mg/kg, dry weight) and magnesium (411.30 mg/kg, dry weight) concentrations in home-produced eggs could be due to the various food’s hens feed at home. On the other hand, the high concentration of phosphorous in market eggs (2318.8 mg/kg, dry weight) may be due to the corn and soybean meal diets for the hens at the farms for egg production. Also, the high concentration of phosphorous in-home eggs (2245.3 mg/kg, dry weight) may be due to whole grains such as millet, whole-wheat bread, rice, and maize [76]. Potassium and calcium contents in home eggs (2331.0 and 839.9 mg/kg) and market eggs (2525.4 and 837.2 mg/kg) are similar to data from the literature [76,77]. Differences in the mineral contents of eggs could be attributed to dietary mineral contents and sources, husbandry systems, and different geographic areas [78].

Heavy metals are released into the environment from both anthropogenic and natural sources with often toxic effects at low concentrations. The heavy metal contamination of food is a severe threat and long-term exposure may lead to human health risks [79]. Chickens are exposed to heavy metals by feed intake, so the metals are passed to humans through chicken eggs [80]. Eggs are a significant source of protein, but if contaminated by heavy metals, they can potentially lead to detrimental effects on human health [81]. The overall mean concentration of aluminum (egg white + yolk) (5.242 mg/kg) was lower than the 17.1 mg/kg reported in some studies [82] and higher than those reported by other studies [83,84], who found that the concentration of aluminum was 4.275 and 2.215 mg/kg, respectively. The mean concentration of this metal for home-grown eggs (4.783 mg/kg) was lower than for commercial eggs (6.927 mg/kg). Factors in the exposure to high-level aluminum would be the dust and feeding hens using grey water, contaminated food, and food cooked in aluminum [85].

Trace elements function in the body as components of enzymes and proteins involved in various biochemical pathways. Zinc (Zn) is known to be an essential constituent in animal nutrition. Therefore, plasma zinc level may indicate egg production or hen performance [86]. A deficiency of Zn causes susceptibility to infections and skin problems, and the excess causes hair loss, weakness, anemia, and stomach and intestine problems [87]. Inappropriate zinc supplementation causes copper deficiency, with this metal having a role in erythrocyte and hemoglobin production and iron absorption in the blood [88]. At the same time, Cu deficiency causes anemia, osteoporosis, and animal depigmentation. Manganese is essential for average growth, skeletal formation, and normal reproductive function in poultry [81]. In normal conditions, the daily ingested amount of manganese is approximately 3–7 mg with a well-balanced diet, with the average concentration of manganese being 0.55 mg/kg in animal ovaries. The sublethal exposure of avian embryos to Mn causes teratogenic effects, such as twisted limbs, hemorrhage, and neck defects [89]. Nickel is a necessary element for humans and animals, with the daily intake of Ni being calculated as 300–600 ppm. A lack of Ni causes growth retardation and anemia; an excess of it causes cancer and toxicity [90].

The mean concentration of zinc in egg white (3.830 mg/kg) was significantly lower than in yolk (33.158 mg/kg); the variation in Zn contents in these parts of the eggs among geographical regions might be due to regional differences of dietary sources or contamination of the environment. The ranges of concentrations of copper and manganese were 0.496–7.828 mg/kg and ND-25.105 in egg white, 1.430–6.074 mg/kg and 0.580–6.27 mg/kg in yolks, and 0.233–1.808 mg/kg and ND-1.772 in eggshells, respectively. The mean concentrations of these elements in home-grown/commercial studied eggs (2.147 and 2.344 mg/kg for Cu; 1.059 and 1.647 mg/kg for Mn; 18.264 and 19.335 mg/kg for Zn, respectively) are in accordance with the results from the literature [10,26,91,92,93].

The experimental results show that the mean concentration of nickel in the studied egg samples (egg white + yolk) was 2.400 mg/kg. Our results are higher for Ni than those of Jagadeesh et al. [7] or Aliu et al. [94] and lower than those of Chowdhury et al. [9]. The high concentration levels of Ni may be related to contamination of the food composition used as hen feed and the activities of repairing and manufacturing industrial workshops near hens’ places [83]. Chromium concentrations in egg samples were recorded within the range of ND (non-detectable) to 160.167 mg/kg, with the highest concentration of chromium being found in a white egg sample. This can be attributed to ingesting food and water containing Cr through processing and preparation by poultry [95]. The chromium content in yolks was determined in concentrations ranging from ND to 33.86 mg/kg. The significantly higher concentration of Cr could be related to the fact that Cr is an essential element and dietary Cr supplementation has been shown to have positive effects on growth performance, egg production, feed efficiency, and egg quality in poultry [96,97]. The mean concentrations of Cr in home-grown/commercial studied eggs (12.389 and 17.306 mg/kg, egg white + yolk) are higher than other data reported in the literature [91,98,99].

Lead (Pb), cadmium (Cd), and mercury (Hg) are carcinogens. They are involved in several diseases such as sclerosis, osteoporosis, developmental disorders, and the failure of organs such as the kidney, lungs, immune system, and heart [100]. The mean contents of Pb, Cd, and Hg in studied eggs were 0.957, 0.023, and 0.121 mg/kg for egg white samples; 1.186, 0.004, and 0.019 mg/kg for yolk samples; and 0.103, 0.004, and 0.0007 mg/kg for eggshell samples, respectively. The high levels of Pb and Cd in the egg samples may be due to natural contamination through feeds or metal contamination in water in their habitat, with food being the primary source of Pb and Cd for humans and animals [101]. The mean levels of Cd and Hg in home-grown eggs/commercial eggs (0.016 and 0.007 mg/kg for Cd; 0.065 and 0.086 mg/kg for Hg, respectively) are more in line with those reported by some authors [10,26] and below those reported by other authors [91,102,103,104].

In addition to the elements discussed above, other elements from different groups were also studied: the group of alkaline and alkaline-earth metals (Li, Cs, Rb, Be, Ba, Sr); rare earth elements (Sc, Y, La, Ce, Pr, Nd, Sm, Eu, Gd, Tb, Dy, Ho, Er, Tm, Yb, Lu); technology-critical elements—TCEs (Te, Ge, Ga, In, Nb, Ta); platinum-group elements—PGEs (Pt, Pd, Rh, Os, Ir, Ru), transition and post-transition metals (Zr, Hf, W, Re, Ti, V, Mo, Bi, Tl); and actinide (Th). The mean concentration of these elements is in accordance with the results from literature [33,63]: 0.477 mg/kg (Li); 0.013 mg/kg (Cs); 3.8 mg/kg (Rb); 0.012 mg/kg (Be); 6.242 mg/kg Ba; 1.945 mg/kg Sr; 0.006 mg/kg (TCE’s); 0.004 mg/kg (PGE’s); 0.513 mg/kg (transition and post-transition metals); 0.023 mg/kg (actinide); and 0.010 mg/kg (REE’s). There are studies reporting that the growth performance in animal production is influenced by the use of REEs as new natural feed additives; thus, these elements are good markers for geographical differentiation [105,106].

### 3.2. Health Risk Assessment

Food safety is a major global concern, mainly because food consumption is a significant pathway of human exposure to heavy metals, accounting for more than 90% [107]. Food contamination is a public health problem worldwide, and the risk associated with exposure to trace metals from food products has aroused widespread concern in human health. Therefore, assessing the heavy metal content in food products and their dietary intake is mandatory to evaluate the health risk.

**Figure 1:**
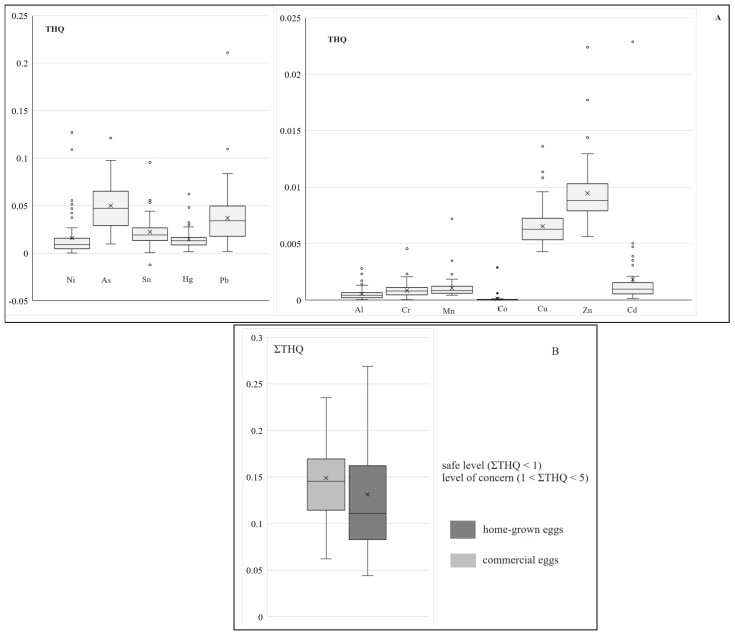
Target Hazard Quotient (THQ) associated with the exposure to different heavy metals detected in the analyzed eggs samples (**A**) and the sum of individual Target Hazard Quotient (**B**). The line across the boxes represents the median value. Whiskers indicate the higher and lower values in the entire data range. The symbols “x” represent the mean markers.

The THQ level associated with the lifetime exposure for adults to metals via chicken egg ingestion is shown in Figure 1A. THQ levels ranged between 0.06 and 0.23 with an average value of 0.14 for the home-grown eggs, and between 0.04 and 0.27 with an average of 0.11 for the commercial eggs (Figure 1B). For all the analyzed samples, the ΣTHQ was below 1, indicating no significant health risks associated with consuming individual heavy metals or a mixture through egg ingestion. Consequently, the non-carcinogenic risk related to the consumption of the analyzed egg samples was in the safe range for the consumers. Studies published in the scientific literature reported similar or lower values for ΣTHQ, for example, 0.008–0.516 for hen eggs collected from Iran [44], 0.032–0.328 for both selenium-enriched and standard egg samples in China [108], and 0.58 for the ingestion of duck eggs from Thailand [107].

### 3.3. Chemometric Results

**Figure 2:**
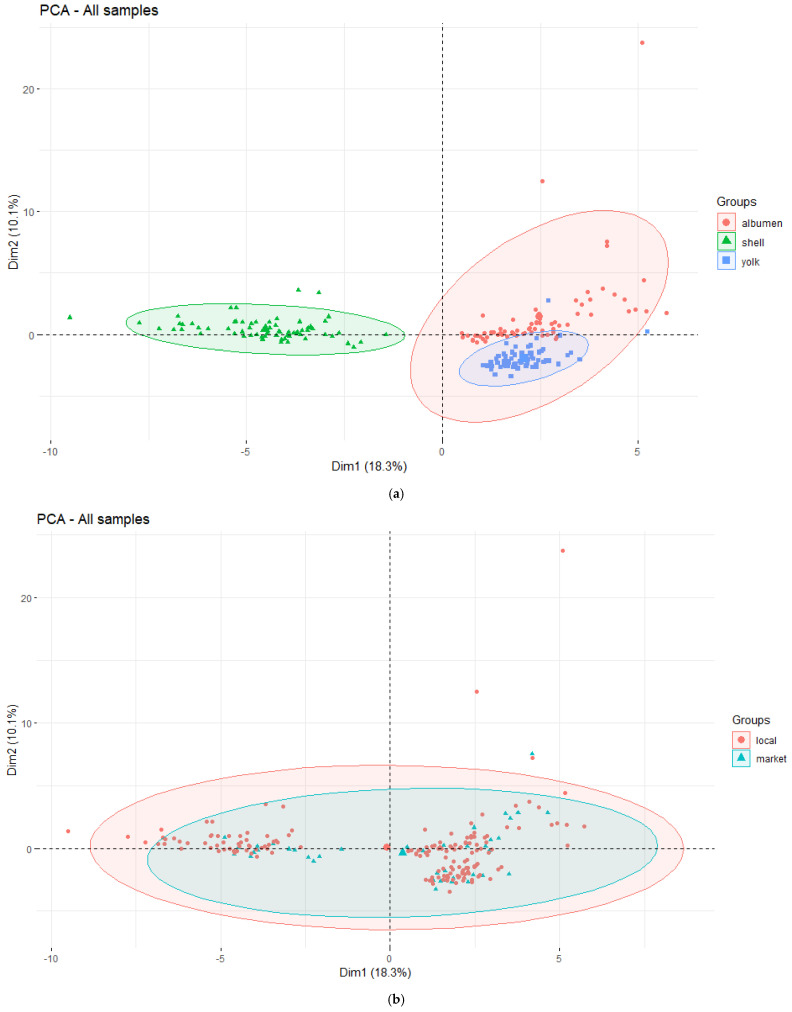

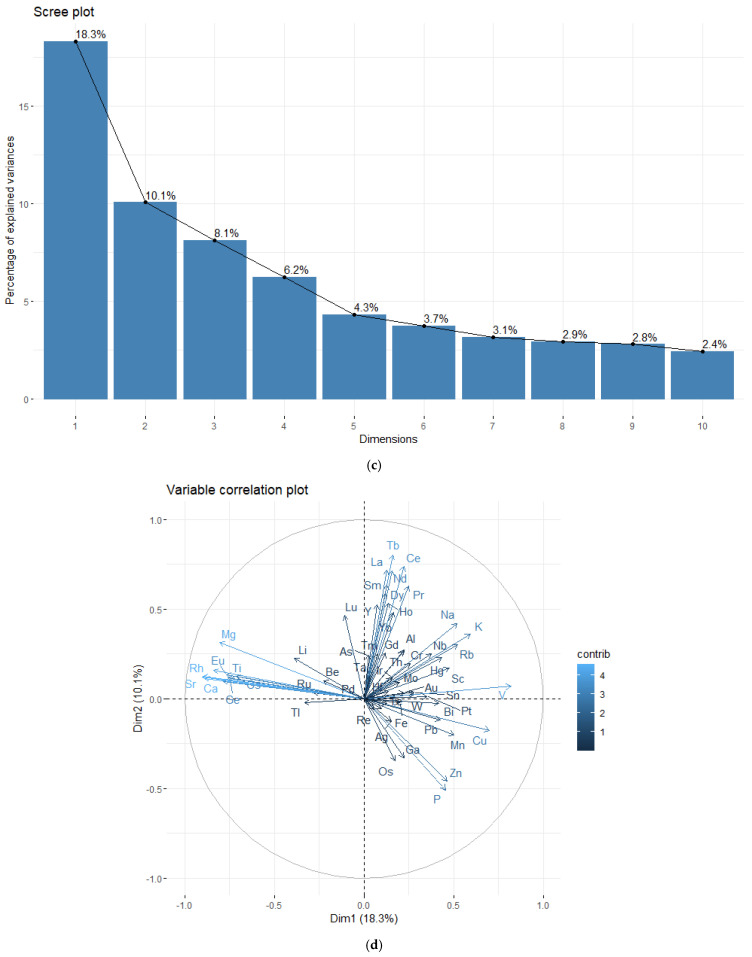
Final PCA results after removing the outliers from the dataset: (**a**) samples plotted on the first two dimensions, grouped based on the egg components; (**b**) rearing growing system of hens; (**c**) scree plot for the first ten dimensions of the final PCA; (**d**) variable correlation plot on the first two dimensions. Grouped variables are positively correlated, while those on opposite sides are negatively correlated. A lighter blue gradient corresponds to a higher variable contribution to the two principal components.

The first two components of the PCA conducted on the trimmed dataset explained a modest 28.4% of the total variance, as visualized in the scree plot (Figure 2). This variance stems from the different chemical compositions of each specific egg component rather than the hens rearing system. The first principal component strongly correlates with observations of the eggshell, while the second main component differentiates between the yolk and albumen. Based on the variable loadings plot, elements Sr, Rh, Ca, Co, V, Mg, Ba, Eu, Ti, Ge, Cs, and Cu contributed most to the first principal component. In contrast, the content of Tb, Ce, La, Nd, Dy, Pr, Sm, and Ho had the highest contribution to the second principal component. Additionally, we can explore other patterns in the data by looking at the variable correlation plot (Figure 2).

**Figure 3:**
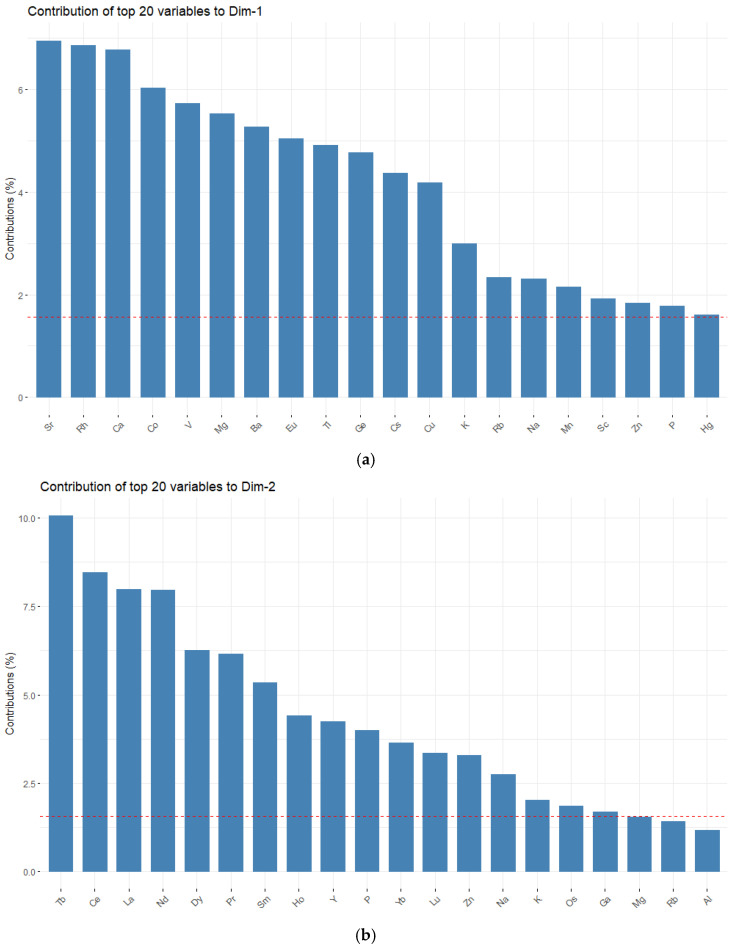
Contributions of the top 20 variables to the first and second components, in decreasing order of strength. The red line indicates the expected average donation of each variable: (**a**) first dimension; (**b**) second dimension.

Among the variables with the highest contribution on the first two principal components, a high positive correlation was observed between Rh, Sr, Ca, Mg, Ti, and Cs. Conversely, Cu and Mg display an almost perfect negative correlation (Figure 3). Tb, Ce, Nd, Dy, and Pr are positively correlated along the second dimension. The PCA must display discernable patterns for differentiating domestic and commercial eggs. With this being the case, we resorted to a PLS-DA with feature selection to achieve this goal.

**Figure 4:**
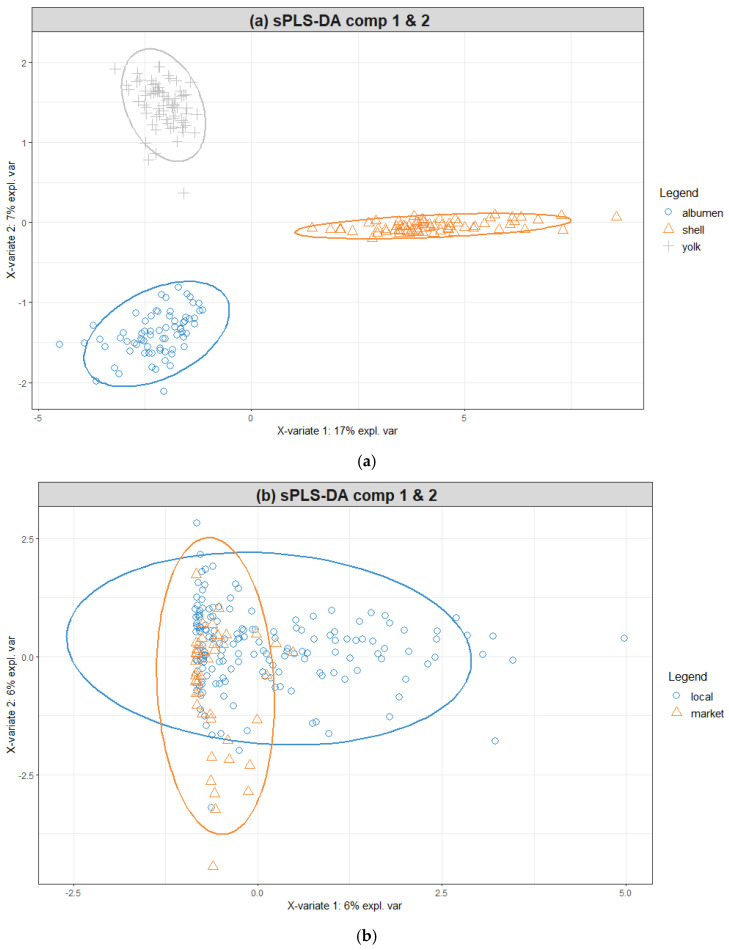
Sample plots using the tuned models for differentiating between (**a**) the components of the egg and (**b**) the hen rearing system. The samples are plotted on the first two dimensions.

The PLS-DA model successfully differentiated between the egg components with a 100% correct classification rate on the test set, using only two components and three variables (Figure 4). Interestingly, the initial, untuned model achieved the same results without the feature selection step. For the tuned model, Ca was the characteristic element for the shell, Na for the albumen, and K for the yolk. This supports our initial principal components analysis results, which indicated that a considerable part of the variance in the dataset came from the unique chemometric fingerprint of each component of the egg we sampled.

**Figure 5:**
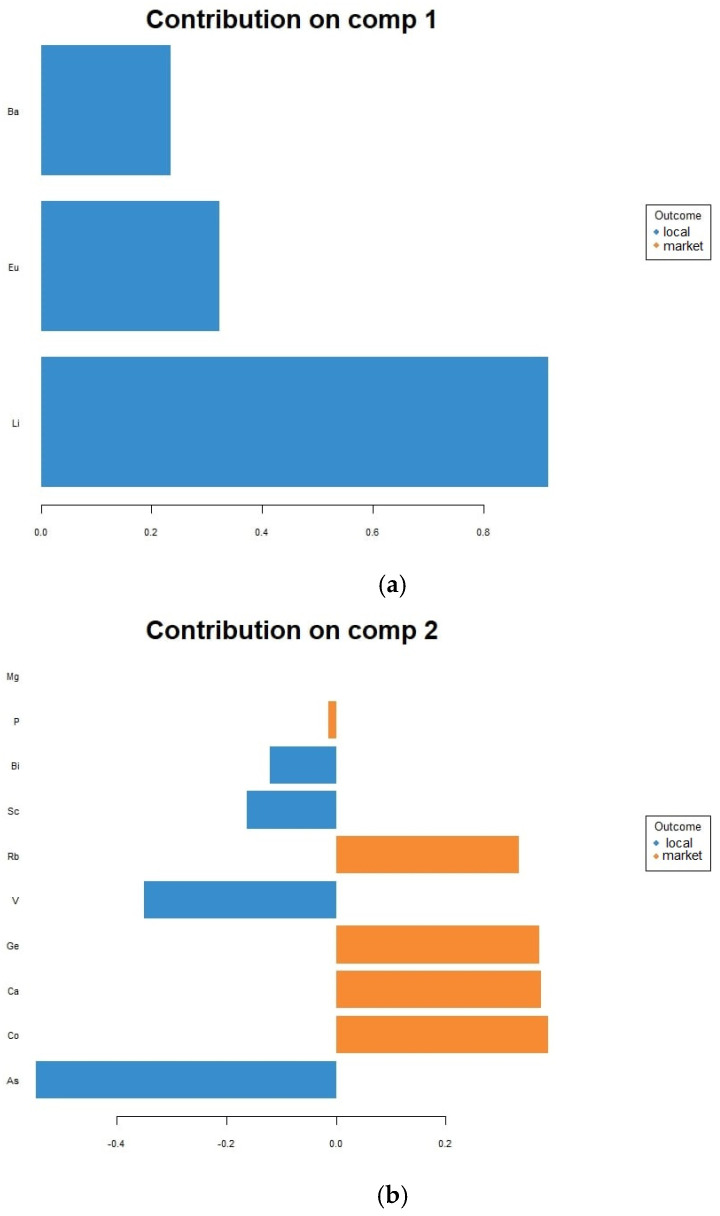

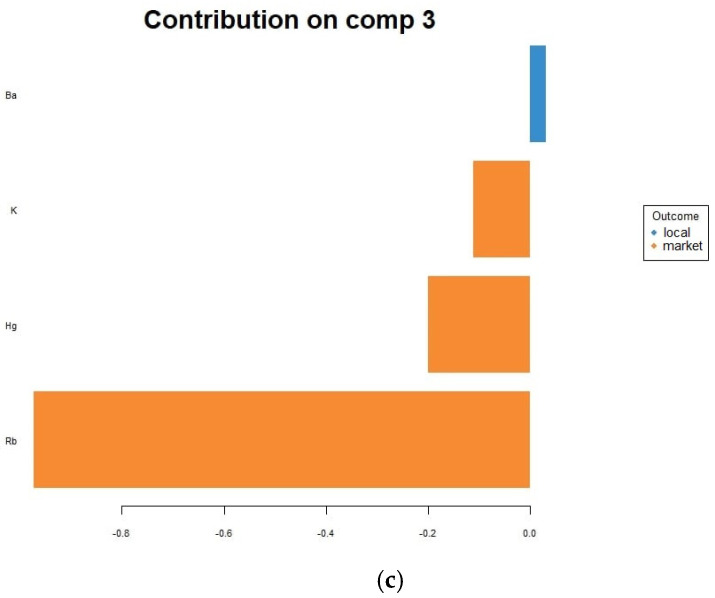
Contributions of elements to the first three components of the final PLS-DA model for differentiating between market and local egg samples: (**a**) first component; (**b**) second component; (**c**) third component.

In the case of identifying the hen rearing system from which the egg comes, the PLS-DA model achieved 80% accuracy, 80% sensitivity, and 85% specificity with three components and 17 variables (Figure 5). Among these variables with the highest discriminatory potential are Li, Eu, and Ba (in decreasing order of strength), whose content contributed the most to the first component, and with Li being strongly associated with the local produced samples. On the second component, As, V, Sc, and Bi contents were associated again with the local egg samples, while Co, Ca, Ge, Rb, and Mg contents were associated with the market samples. Lastly, Rb was strongly linked with the market eggs on the third component, followed by Hg and K. Ba only had a mild association with the local egg samples.

## 4. Conclusions

Chicken eggs are one of the primary sources of protein, but contamination by toxic heavy metals due to industrial waste, geochemical structures, and agricultural activities is a severe problem for human health.

Heavy metal contamination of eggs is a significant problem for public health, having several acute and long-term harmful effects on different human organs. Either during production or consumption, heavy metals can taint eggs, through chicken feed and drinking water, both of which are largely influenced by the environment. This pollution can directly or indirectly affect human health through contamination of the food chain. The Romanian food system heavily depends on egg or meat chickens, and their contamination can have important effects; therefore, this study was conducted with a special look at hen eggs as one of the most Romanian consumed foods.

Romanian egg quality was assessed to establish the possible risk to human health using heavy metals concentrations, due to their cumulatively negative impact on human health.

Five essential elements and the trace element concentrations were measured in a total set of 210 samples, formed from the component parts (egg white, yolk, and shell) of 70 eggs, comprising 55 home-produced eggs and 15 commercial eggs. The range of variation in mineral content in egg samples presented different values, depending on the rich nutrition system of hens and other factors such as the impact of environmental and physiological reasons and husbandry practices. Regarding food safety, the toxic element concentrations in most investigated samples were below the maximum permitted levels set by Romanian and European legislation. For all the samples, the Target Hazard Quotient sum for each heavy metal was below 1, indicating no significant health risks associated with consuming individual heavy metals or a mixture through egg ingestion.

PLS-DA analysis highlights the potential of chemometric fingerprinting to differentiate egg components and rearing systems. Perfect performance was achieved when distinguishing between egg parts, which was to be expected given the characteristic chemical composition associated with the yolk, albumen, or shell. In addition, solid discriminative performance was demonstrated when comparing supermarket and domestic samples using 17 elemental markers.

To ensure the safety of hen eggs produced for consumer use, more research on heavy metal contamination in hen organs and eggs should be conducted. It is advised that future studies evaluate the presence of heavy metals in chicken feed, water, and meat on an individual basis. Contamination of eggs with heavy metals is a significant public health problem, having several acute and long-term harmful effects on various human organs. Either during production or during consumption, heavy metals can contaminate eggs, through chicken feed and drinking water, both of which are heavily influenced by the environment, and pollution can directly or indirectly affect human health by contaminating the food chain.

Due to budget limitations, a certain number of samples were analyzed in this study, and the studied samples may not be representatives of all eggs in Romania. This study presents the heavy metal content in eggs and does not necessarily reflect the consumption of other products, which could introduce heavy metals into the body. As a result, it is advised that heavy metal levels in hen eggs and other foods in Romania be routinely monitored.

The data gathered in this study could provide significant tools for upcoming ecotoxicological inquiries and biosafety in implementing regulations and standards for commercial hen egg production.

## Figures and Tables

**Table 1:** Macro-element concentrations in egg components (dry weight, mg/kg).

Elements	Element Concentration (mg/kg)			
	Egg	Minimum	Maximum	Mean
**Na**	egg white	5900.00	15,373.56	10,266.37
	yolk	156.75	1200.11	629.06
	eggshell	300.22	1183.84	653.64
**Mg**	egg white	290.55	965.85	639.20
	yolk	53.10	230.45	176.03
	eggshell	738.57	3000.70	1488.01
**P**	egg white	220.38	1400.02	665.93
	yolk	1331.11	5000.44	3856.23
	eggshell	230.29	873.81	521.04
**K**	egg white	526.95	6977.12	4151.84
	yolk	280.72	921.95	593.43
	eggshell	85.56	346.10	156.72
**Ca**	egg white	130.11	2928.81	453.49
	yolk	438.15	1843.34	1225.18
	eggshell	40,813.66	139,519.80	74,643.56

## Data Availability

Data are contained within the article.
